# Transforming the odor profile of perennial ryegrass protein and surplus bread crusts through solid-state fermentation

**DOI:** 10.1038/s41538-026-00831-6

**Published:** 2026-04-24

**Authors:** Juan F. Sandoval, Jane K. Parker, Joe Gallagher, Julia Rodriguez-Garcia, Kerry Whiteside, David N. Bryant

**Affiliations:** 1 Institute of Biological, Environmental and Rural Sciences (IBERS), Aberystwyth University, Aberystwyth, UK; 2 Department of Food and Nutritional Sciences, University of Reading, Reading, UK; 3 Nutrition and Food Science Area, Preventive Medicine and Public Health, Food Science, Toxicology and Forensic Medicine Department, Faculty of Pharmacy, Avda. Vicent Andrés Estellés, Universitat de València, Burjassot, Spain; 4 Samworth Brothers Limited, Leicestershire, UK

**Keywords:** Biochemistry, Biological techniques, Biotechnology, Microbiology, Plant sciences

## Abstract

The growing demand for sustainable, high-quality protein sources is driving the development of novel foods. In this study, the underexplored combination of surplus bread crusts (BC), a major source of food waste, and perennial ryegrass (PRG), a traditional forage crop with underexploited potential for human nutrition, was used as a potential substrate in the production of protein-rich novel ingredients for human consumption. The BC substrate supplemented with crude PRG protein was fermented with *Rhizopus oligosporus*, *Aspergillus oryzae* or *Neurospora intermedia* for up to 72 h to enhance the nutritional and olfactory qualities biologically. After solid-state fermentation (SSF), the volatile organic compounds (VOCs) that were present were captured by solid-phase microextraction followed by gas chromatography-mass spectrometry. Over 150 compounds were identified at different stages of SSF and correlated to specific fungi or substrates via principal component analysis (PCA). These results provide an understanding of the composition of VOCs throughout fungal SSF and prove its capacity to improve the odor of forage crops for novel food production.

## Introduction

Efforts to develop sustainable methods for converting food waste and plant biomass have driven advances in the production and improvement of alternative protein sources for human consumption. Biological processing methods, namely solid-state fermentation (SSF), have been proven to nutritionally enhance the biomass of cereals, legumes, fruits, and vegetables^1^. Surplus wheat bread, one of the most common sources of food waste globally^2^, has been valorized via SSF with filamentous fungi, increasing the content of crude protein, crude fiber, essential amino acids, minerals, and vitamins^3,4^.

Aside from compositional changes, the flavor and odor profiles of the substrates are altered by SSF. Several authors have studied the traditional fermentation of wheat flour doughs with *Saccharomyces cerevisiae* and lactic acid bacteria, a prime example of SSF, identifying over 190 volatile organic compounds (VOCs) responsible for its complex and characteristic sensory profile^5^, of which at least 40 are affected during the fermentation^6^. Other applications of SSF have also targeted the production of odor-active compounds from traditional cereals and legumes, such as wheat, rice, and soybean, with microorganisms such as *Aspergillus oryzae*, *Bacillus* sp., and *Neurospora sp.*^7–10^. Additionally, SSF has been applied to agro-industrial wastes such as bagasse, brans, pomaces, husks, and oil cakes from diverse origins with *Aspergillus niger, Ceratocystis fimbriata, Fomitopsis pinicola*, *Kluyveromyces marxianus, Rhizopus oryzae, Moniliella suaveolens, Saccharomyces cerevisiae,* and *Trichoderma harzianum*^11–13^. Notably, different microorganisms induce distinct changes in the production or reduction of specific odor-active compounds, which are also influenced by changes in the substrate.

The changes in the flavor and odor profile induced by SSF can potentially make alternative feedstocks rich in high-quality protein, previously disregarded due to unfavorable sensory characteristics, a new resource for the manufacture of novel ingredients and foods. One highly available but underexploited source of protein, outside of livestock production, is forage crops, such as perennial ryegrass (*Lolium perenne*) (PRG), which has a high crude protein (CP) content (22.6% dry matter (DM))^14^, a well-balanced amino acid profile^15^, and is rich in minerals^16^ and nutraceutical compounds^17^. However, besides the high fiber content (50.9% DM)^14^, it has a characteristic green and grassy flavor and odor profile, making it unfit for direct food applications. Purified protein extracts from these crops have been studied for food applications^18^, but human sensory trials of these materials are not common^19^.

SSF of grass protein-rich juice extracts with filamentous fungi could alter their characteristic odor profile, but requires another substrate to function as a scaffold. It has been proven that PRG protein can be combined with surplus bread and fermented with *Rhizopusg/oligosporus*, improving the crude protein and essential amino acid content of the combined substrate^20^. Such a process would also modify the odor profile, a complex mixture of VOCs from PRG, surplus bread, and fungal metabolism.

Important VOCs that give grasses their characteristic odor profiles have been identified as a means to understand their relationship with silage quality^21,22^, animal performance^23,24^, and air quality^25^. These include sulfur-containing compounds (e.g., 2-methylthiophene, methional and dimethyl sulfide) with green, sulfurous and vegetable notes, esters (e.g., ethyl octanoate, ethyl acetate and ethyl hexanoate) with diverse sweet, fruity and alcoholic notes, aldehydes (e.g., 2-methylbutanal, 3-methylbutanal, methylpropanal, benzaldehyde and phenylacetaldehyde) with diverse green, malty and floral notes, alcohols (e.g., hexanol, 2-hexanol and (*Z*)-3-hexenol) and terpenes (e.g., linalool, citronellol, isogeraniol, eugenol and limonene) with varied herbal, woody, floral and spiced notes. The resulting odor profile is complex based on the different concentrations of these compounds, their odor thresholds, and complex chemical interactions. The odor of many of these compounds is mainly known from their presence in other sources, such as cereals, legumes, fruits, and vegetables^26^, but they have not been studied in grasses in relation to food applications.

Overall, previous research has identified the main VOCs that make up the odor profile of grasses and bread. While the changes induced by fungal metabolism during SSF of different substrates have been reported^5,8,27,28^, the effect of SSF on the odor profile of grasses or other forage crops is unknown. Thus, this study aimed to identify the VOCs present after different times of SSF of surplus bread supplemented with PRG with *Aspergillus oryzae* (AO)*, Neurospora intermedia* (NI), or *Rhizopus oligosporus* (RO), and is the first report of its kind in the literature. This investigation tested the hypothesis that different species of filamentous fungi would induce specific changes in the odor profile of the mixed substrate at different SSF times, which, aside from reducing grass-related notes, would induce positive notes that would have potential applications for the development of novel ingredients and foods.

## Results and discussion

### Solid-state fermentation

Surplus bread crusts (BC) are an ideal substrate for SSF due to their richness in carbohydrates and the porosity of their structure^29^, made up of networks of gluten (15% of the dry matter (DM) content of bread) and starch (71% of the DM content)^30^. While it has been proven that filamentous fungi such as *Neurospora* sp. and *Rhizopus* sp. can successfully grow in this substrate to increase the CP, crude fiber, and essential amino acids content^3,4^, the amino acid profile of gluten does not possess all of the amino acids required by an average adult. This requirement is defined by the Food and Agriculture Organization of the United Nations (FAO) guidelines^31^, which justifies the supplementation of the substrate with other sources of protein that complement the profile. This supplementation has been done with PRG protein, in the form of grass juice extracts (GJ) and dried grass protein solids (GS), increasing the content of amino acids found in low concentrations in gluten, such as lysine, methionine, and cysteine^20^. This work identified that the optimal supplementation of PRG protein in BC was up to a final CP content of 27% DM.

The SSF experiments were carried out in both BC + water (B + W) and BC + PRG (B + G) substrates at different SSF times and with AO, NI and RO to (1) facilitate the identification of the origin of the volatile compounds from either BC, PRG or fungal metabolism, (2) study the differences in SSF of BC over the VOCs in the presence of PRG and (3) study the differences between filamentous fungi in the volatile composition. The composition of BC, GJ, and GS, and the unfermented mixed substrates B + W and B + G can be seen in Table 1.

**Table 1:** Composition of unfermented solid-state fermentation (SSF) substrates

Material	H (%)	CP (%DM)
Surplus bread crusts	20.8 ± 0.5%	16.8 ± 0.2%
Grass juice	94.9 ± 0.4%	16.0 ± 0.7%
Dry grass solids	15.0 ± 0.5%	40.1 ± 0.5%
Bread crusts + water	56.0%*	16.8%*
Bread crusts + perennial ryegrass	56.0%*	27.0%*

Data are shown as the mean value and a standard error.

*Values from the mass balance of components.

*H* Moisture, *CP* Crude protein, *DM* Dry matter.

**Fig. 1:**
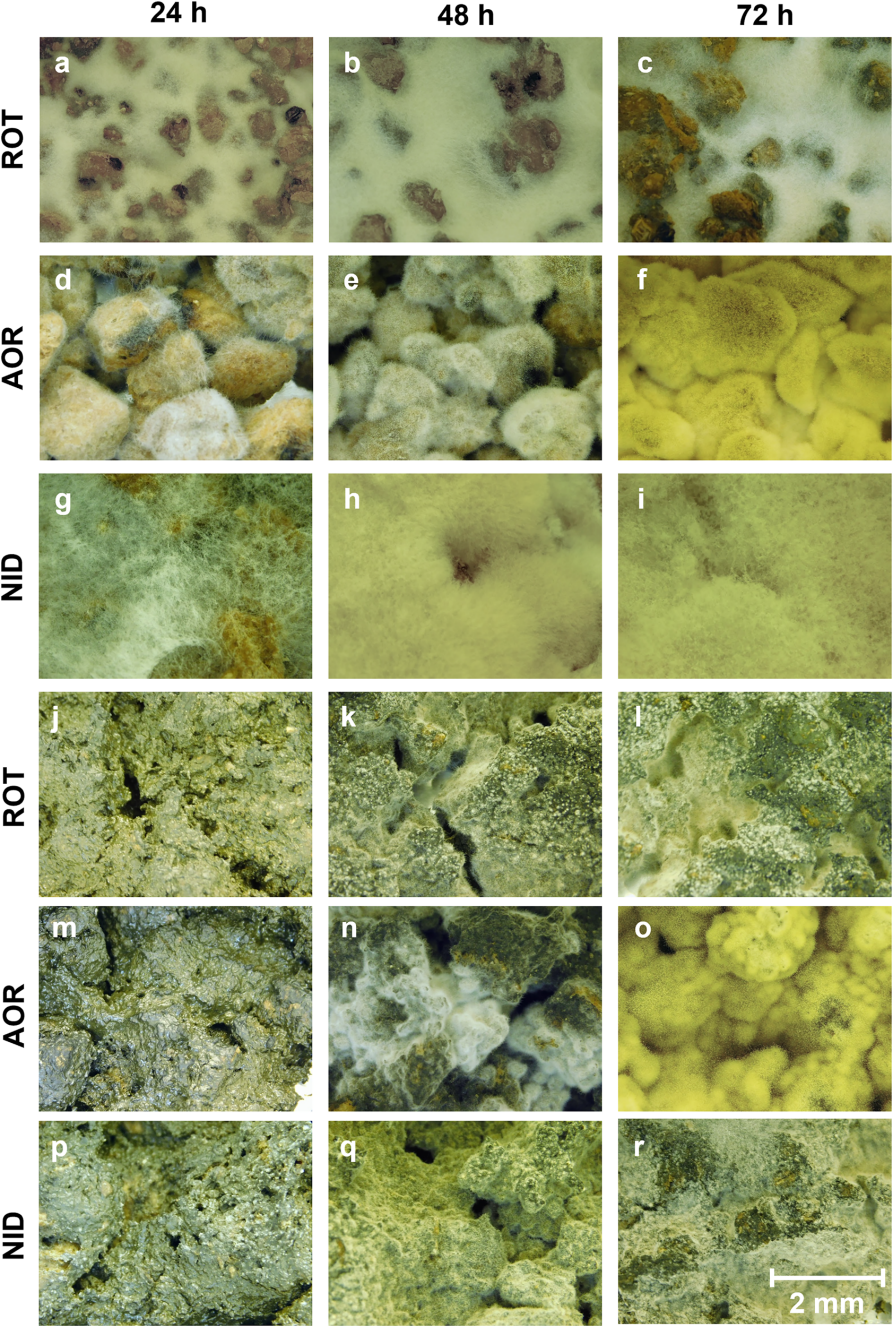
Changes during solid-state fermentation (SSF). Photographic record of SSF experiments for *Rhizopus oligosporus* (Tempeh) (ROT), *Aspergillus oryzae* (Red rice koji) (AOR), and *Neurospora intermedia* (DSMZ 1265) at 24, 48, and 72 h of fermentation in bread crusts + water (B + W) and bread crusts + perennial ryegrass (B + G) substrates. ROT in B + W **a** 24 h, **b** 48 h, **c** 72 h. AOR in B + W **d** 24 h, **e** 48 h, **f** 72 h. NID in B + W **g** 24 h, **h** 48 h, **i** 72 h. ROT in B + G **j** 24 h, **k** 48 h, **l** 72 h. AOR in B + G **m** 24 h, **n** 48 h, **o** 72 h. NID in B + G **p** 24 h, **q** 48 h, **r** 72 h.

A photographic record of the growth of some of the fungi can be seen in Fig. 1. For all six strains used in the fermentations, the growth was faster in the B + W substrate, where, after 24 h of SSF, the mycelia had completely covered the BC. For all strains, sporulation began to occur by 72 h, as evidenced by changes in the color of the mycelia, which represent the characteristic color of the spores of each fungus (black/deep brown for RO, green/yellow for AO, and pink for NI). Understanding sporulation as a biological marker is important in the context of VOC generation in SSF, as it has been shown that VOC concentrations increase at the onset of sporulation, positively or negatively affecting the odor profile depending on the intended application. *Aspergillus sp*. and *Penicillium sp*. grown in oatmeal agar produced multiple VOCs with unpleasant musty odors, such as dimethyl disulfide, 1-octen-3-ol, 2-methylisoborneol, and geosmin, and others with pleasant odors such as 3-methylfuran and 2-methylpropanol, and their concentrations spiked at the time of sporulation^32^.

In the B + G substrate, RO and NI did not grow as well as AO, which also grew at a slower initial rate when comparing its growth at 24 and 48 h. By 72 h, all fungi achieved at least superficial mycelial coverage of the substrate, but RO and NI did not reach sporulation. The slower growth in this substrate was likely a consequence of the phenolic compounds found in PRG, which have been proven to have antifungal properties^20^.

**Fig. 2:**
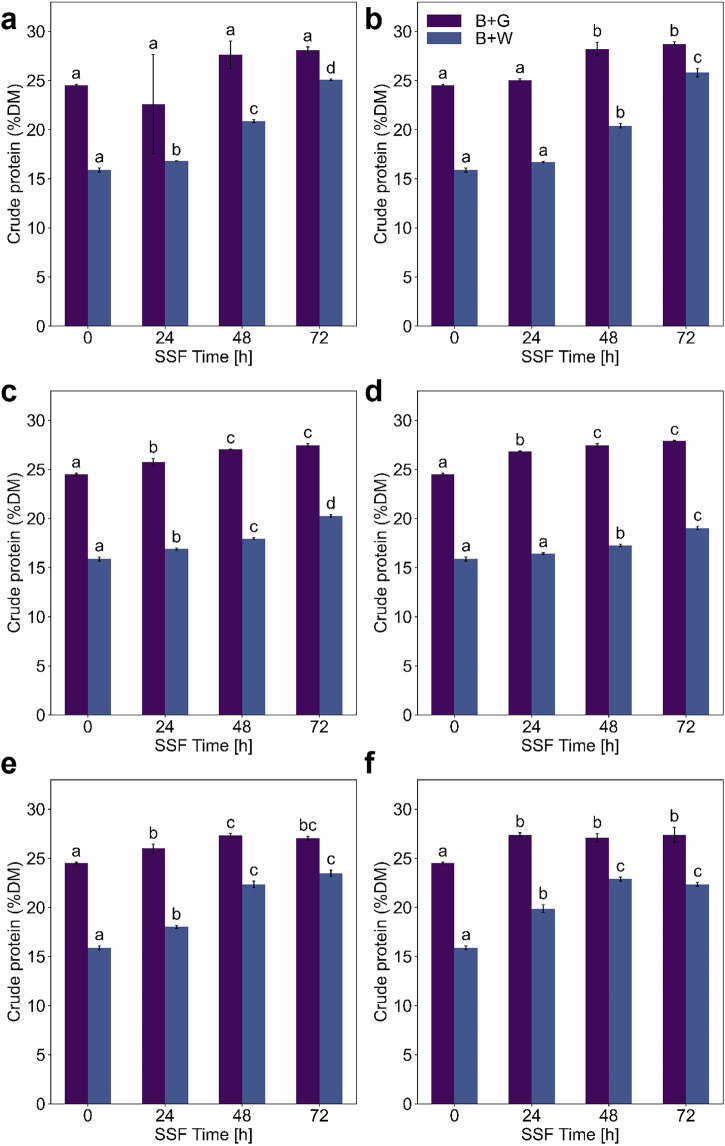
Crude protein content of of solid-state fermentation (SSF) samples. Crude protein (% dry matter) at 0, 24, 48, and 72 h of SSF with **a** *Aspergillus oryzae* (Barley koji) (AOB). **b** *Aspergillus oryzae* (Red Rice koji) (AOR). **c** *Neurospora intermedia* (CBS 131.92) (NIC). **d** *Neurospora intermedia* (DSMZ 1265) (NID). **e** *Rhizopus oligosporus* (DSMZ 1964) (ROD). **f** *Rhizopus oligosporus* (Tempeh) (ROT), in bread crusts + water (B + W) and bread crusts + perennial ryegrass (B + G) substrates. Bars represent the mean value (*n* = 3) and vertical lines the standard error. Data with the same letters in the substrate group are not significantly different (*p* = 0.05), following Tukey´s HSD post-hoc test.

The resulting CP composition of the B + W and B + G substrates after different times of SSF with the different fungi can be seen in Fig. 2. In all cases, CP content increased progressively through SSF time as a result of the metabolic activity of the fungi. In B + W substrates, the maximum CP content was achieved for both AO and NI SSF strains at 72 h, while for RO, this happened after 48 h. In B + G substrates, the maximum CP content was achieved for most fungi after 48 h of SSF, except for ROT, which was attained after 24 h.

### Identification of volatile compounds

A total of 156 VOCs were identified across all SSF experiments (Table 2). The compounds were classified according to their functional chemical group for a total of 17 alcohols, 25 aldehydes, 21 esters, 11 furans, 14 ketones, 4 organic acids, 4 phenols, 9 pyrazines, 1 pyridine, 6 pyrroles, 5 sulfur compounds, 28 terpenes, 7 dioxolanes, and 4 compounds that did not fit the other groups. When classified by their likely source of origin, there were 29 products typical of lipid oxidation, 41 typical of Maillard reactions, 32 likely to be derived from PRG, 49 likely to have been produced during SSF, and 5 that did not fit the other groups (Data S1).

**Table 2:** Volatile compounds in solid-state fermented (SSF) surplus bread crusts and perennial ryegrass with *Aspergillus oryzae*, *Neurospora intermedia*, and *Rhizopus oligosporus*

n.	Compound name	LRIᵃᵇ	Odor descriptorsᶜ	IDᵈ
**Alcohols**				
1	2-methylpropanol	623	ethereal, bread, fermented	A
2	butanol	659	fusel	A
3	1-penten-3-ol	680	horseradish	A
4	3-methylbutanol	733	alcoholic, banana	A
5	2-methylbutanol	736	chocolate	A
6	pentanol	765	fusel, green	A
7	2,3-butanediol (isomer 1)	789	butter	A
8	2,3-butanediol (isomer 2)	793	butter	A
9	*(Z)-*3-hexen-1-ol	851	green	A
10	2-hexen-1-ol	866	green	A
11	hexanol	868	green, fatty	A
12	heptanol	969	fusel, green	A
13	2-octanol	999	floral	A
14	benzyl alcohol	1038	floral	A
15	octanol	1070	waxy	A
16	phenylethyl alcohol	1120	floral	A
17	*(E)-*3-nonen-1-ol	1171	green, cucumber	B
**Aldehydes**				
1	methylpropanal	562	cocoa, malty	A
2	2-butenal	648	chocolate	A
3	3-methylbutanal	651	cocoa, malty	A
4	2-methylbutanal	661	cocoa, malty	A
5	pentanal	698	fermented, cocoa	A
6	2-methyl-2-butenal	743	pungent, nutty	A
7	*(E)-*2-pentenal	753	fatty, fruity	A
8	3-methyl-2-butenal	785	almond	A
9	hexanal	800	green	A
10	*(Z)-*3-hexenal	800	green	A
11	*(E)-*2-hexenal	854	green	A
12	*(Z)-*4-heptenal	900	linseed	A
13	*(E)-*2-heptenal	958	fatty	A
14	phenylacetaldehyde	1050	floral	A
15	*(E)-*2-octenal	1061	fruity, fatty	A
16	5-methyl-2-isopropyl-2-hexenal	1092	green, chocolate	B
17	nonanal	1106	fruity, fatty	A
18	*(E)-*5-methyl-2-isopropyl-2-hexenal	1112	green, floral	A
19	*(E,E)-*2,6-nonadienal	1157	waxy, cucumber	A
20	*(E,Z)-*2,6-nonadienal	1163	waxy, cucumber	A
21	*(E)-*2-nonenal	1167	fatty	A
22	*(E)-*2-decenal	1266	cilantro	A
23	2-phenyl-2-butenal	1283	musty, sweet, narcissus, beany, nutty, radish	B
24	*(E)-*2-undecenal	1368	cilantro	A
25	5-methyl-2-phenyl-2-hexenal	1503	cocoa	A
**Esters**				
1	ethyl acetate	613	ethereal	A
2	ethyl lactate	798	fruity	A
3	propyl propanoate	806	fruity	A
4	ethyl 2-butenoate	844	fruity	A
5	ethyl hexanoate	998	fruity	A
6	2-hexenyl acetate	1018	fruity	A
7	ethyl *(E)-*2-hexenoate	1044	aniseed, sweet	A
8	2-methylbutyl butanoate	1059	fruity	A
9	ethyl heptanoate	1097	pineapple	B
10	ethyl 4-octenoate	1190	fruity	B
11	ethyl octanoate	1196	fruity	A
12	ethyl 3-octenoate	1198	fruity	B
13	ethyl *(E)-*2-octenoate	1246	fruity	B
14	2-phenethyl acetate	1263	coconut	A
15	α-terpinyl acetate	1353	herbal	A
16	neryl acetate	1359	floral	A
17	2-phenylethyl propanoate	1360	sweet	B
18	ethyl *(Z)-*4-decenoate	1380	fruity	B
19	ethyl decanoate	1394	fruity, fatty	A
20	citronellyl butanoate	1534	fruity, floral	A
21	geranyl butanoate	1562	floral	A
**Furans**				
1	2-methylfuran	606	ethereal, chocolate	A
2	3-methylfuran	638	mold	A
3	2-ethylfuran	703	burnt sugar	A
4	2-ethenylfuran	725		A
5	2-furfural	835	almond	A
6	2-furanmethanol	860	caramel, rum	A
7	5-methyl-2(3*H*)-furanone	870	green, musty	A
8	2-methyl-5-propylfuran	888	vegetable	B
9	2-acetylfuran	914	cocoa, coffee	A
10	5-methyl-2(5*H*)-furanone	940	sweet, maple	B
11	2-pentylfuran	994	mushroom, fatty, beany	A
**Ketones**				
1	2,3-butanedione	589	butter	A
2	2-butanone	599	ethereal	A
3	3-methyl-2-butanone	656	solvent	A
4	1-pentene-3-one	686	fishy, tomato	A
5	2,3-pentanedione	695	butter	A
6	3-hydroxybutanone	710	butter	A
7	4-cyclopentene-1,3-dione	887		A
8	2-heptanone	891	pear	A
9	2-methyl-2-cyclopentenone	907	caramel, maple	A
10	3-methyl-4-heptanone	929	fruity, hazelnut	B
11	6-methyl-2-heptanone	956	fruity	A
12	2,3-octanedione	984	aldehydic, green	A
13	6-methyl-5-heptene-2-one	988	fruity	A
14	2-hydroxy-3-methyl-2-cyclopenten-1-one	1029	burnt sugar	A
**Organic acids**				
1	acetic acid	597	vinegar	A
2	2-methylpropanoic acid	750	cheesy	A
3	3-methylbutanoic acid	833	cheesy	A
4	2-methylbutanoic acid	846	cheesy	A
**Phenols**				
1	4-ethenylphenol	1219	medicinal	A
2	4-ethenyl-2-methoxyphenol	1324	smokey	A
3	eugenol	1368	clove	A
4	1,2-dimethoxy-4-prop-1-enylbenzene	1501	clove	A
**Pyrazines**				
1	methylpyrazine	826	marzipan	A
2	2,5-dimethylpyrazine	916	nutty	A
3	ethylpyrazine	918	cocoa, bread	A
4	2,3-dimethylpyrazine	922	nutty, biscuit	A
5	2-isopropylpyrazine	972	baked	A
6	2-ethyl-5-methylpyrazine	1001	sweet, nutty	A
7	2,6-diethylpyrazine	1083	nutty, biscuit	A
8	3,6-dimethyl-2-ethylpyrazine	1083	roasted, earthy	A
9	3,5-diethyl-2-methylpyrazine	1163	nutty, roasted	B
**Pyridines**				
1	2-acetylpyridine	1038	toasty	A
**Pyrroles**				
1	1-ethylpyrrole	816	meaty	A
2	2-pyrrolecarboxaldehyde	1008	beefy	A
3	1-ethyl-2-pyrrolecarboxaldehyde	1057	burnt	B
4	2-acetylpyrrole	1062	bread crust	A
5	1-(3-methylbutyl)pyrrole	1063		A
6	1-(2-furanylmethyl)-1*H*-pyrrole	1189	vegetable, earthy	A
**Terpenes**				
1	α-phellandrene	1011	herbal, medicinal	A
2	p-cymene	1031	herbal	A
3	D-limonene	1036	lemon	A
4	2,2,6-trimethylcyclohexanone	1042	floral	B
5	terpinolene	1095	pine	A
6	linalool	1101	citrus, lavender	A
7	α-cyclocitral	1128	fruity, floral	A
8	α-terpineol	1200	terpenic, lime	A
9	safranal	1211	herbal	A
10	β-cyclocitral	1233	fruity, medicinal	A
11	β-cyclohomocitral	1271	camphoraceous	A
12	dihydroedulan	1306		B
13	α-copaene	1390	woody	A
14	α-santalene	1426	woody	A
15	caryophyllene	1444	woody, medicinal	A
16	6,10-dimethyl-5,9-undecadien-2-one	1458	floral	A
17	*(E)-*β-farnesene	1462	terpenic, peel, apricot	A
18	β-chamigrene	1475		B
19	α-humulene	1478	hops	A
20	β-curcumene	1492	spicy	B
21	α-cuparene	1495		B
22	dehydro-β-ionone	1497	orris	B
23	β-bisabolene	1507	woody	A
24	*(Z)-*α-bisabolene	1513	woody	A
25	cubebol	1521	pepper	A
26	dihydroactinolide	1535	coumarin, musk	B
27	*(Z)-*nerolidol	1538	floral, citrus	A
28	santalol	1697	woody	A
**Sulfur compounds**				
1	dimethyl sulfide	534	seaweed	A
2	dimethyl disulfide	747	vegetable	A
3	methional	908	potato	A
4	5-methyl-2- thiophenecarboxaldehyde	1131	almond, bread	A
5	3-methyl-2- thiophenecarboxaldehyde	1135	meaty, roasted	A
**Dioxolanes**				
1	4,5-dimethyl-2-isopropyl-1,3-dioxolane (isomer 1)	876		A
2	4,5-dimethyl-2-isopropyl-1,3-dioxolane (isomer 2)	912		A
3	4,5-dimethyl-2-isopropyl-1,3-dioxolane (isomer 3)	920		A
4	4-methyl-2-(2-methylpropyl)-1,3-dioxolane	952	Fruity	A
5	4,5-dimethyl-2-isobutyl-1,3-dioxolane (isomer 1)	975		A
6	4,5-dimethyl-2-isobutyl-1,3-dioxolane (isomer 2)	1005		A
7	4,5-dimethyl-2-isobutyl-1,3-dioxolane (isomer 3)	1015		A
**Others**				
1	p-xylene	880	burnt plastic	A
2	nonane	899		A
3	benzaldehyde	966	almond	A
4	1,1,6-trimethyl-1,2-dihydronaphthalene	1372	petrol	A

ᵃLinear retention index on a DB-5 column, calculated from a linear equation between each pair of straight chain alkanes C6–C20.

ᵇComparison of LRIs and their source can be found in Data S1.

ᶜOdor descriptors identified by smelling in 1% ethanol solutions.

ᵈA: Mass spectra and authentic LRI match, B: Mass spectra and external LRI match.

The CAS number for each of the compounds can be found in the Data S1.

**Fig. 3:**
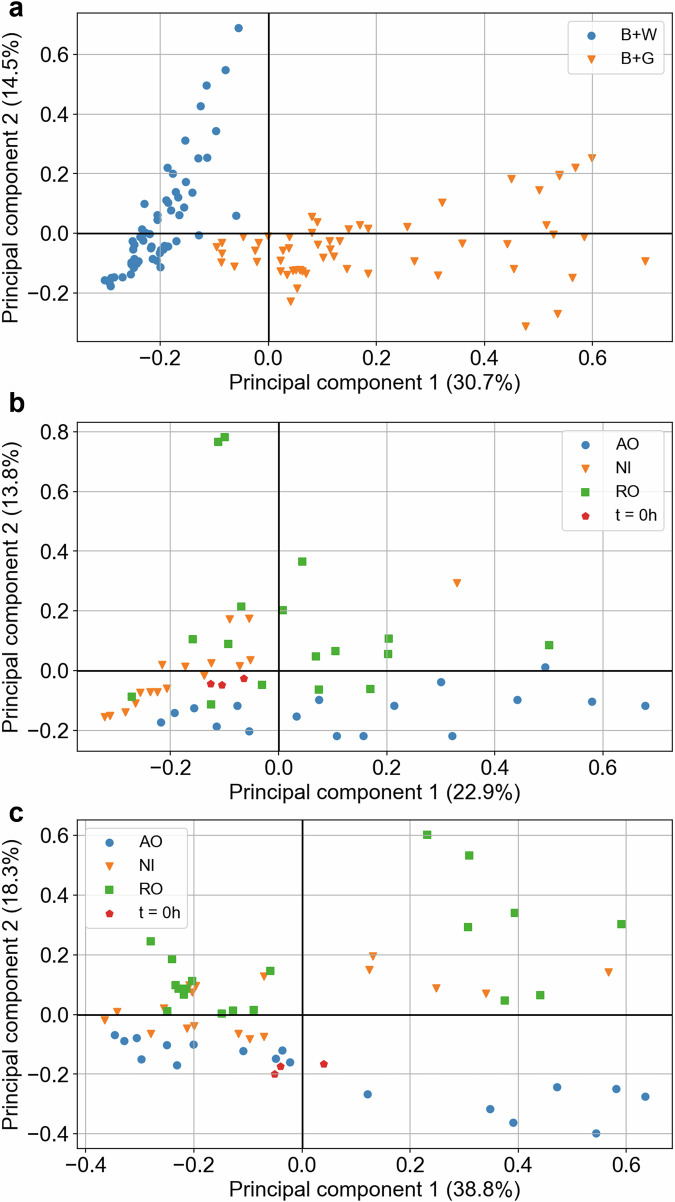
Principal component analysis (PCA) of volatile organic compound (VOC) profiles. PCA of VOCs at 0, 24, 48, and 72 h of solid-state fermented (SSF) samples detected by GC-MS. **a** For all samples, grouped by substrate as either bread crusts + water (B + W) or bread crusts + perennial ryegrass (B + G). **b** For fermentations in B + W substrate, grouped by fungi as either *Aspergillus oryzae* (AO), *Neurospora intermedia* (NI), or *Rhizopus oligosporus* (RO), and unfermented substrate (*t* = 0 h). **c** For fermentations in B + G substrate, grouped by fungi as either AO, NI, or RO, and unfermented substrate (t = 0 h).

MANOVA was carried out on all identified VOCs in each combination of fungi, substrate, and SSF time, finding significant variances in many of them (*p*-value < 0.05). Due to the high number of VOCs, PCA was used to understand their correlation and aid in visualizing the differences between the experimental points (Data S2). Initially, PCA was performed over all samples (Fig. 3a), which showed a clear grouping based on the SSF substrate as either B + W or B + G. Based on these components, VOCs with positive weights on PC1 are related to B + G, and VOCs with negative weights in PC1 are associated with B + W, mostly originating from the BC. Those with weights close to 0 are related to both BC and PRG, as many VOCs were found to be naturally occurring in both substrates. This aids in identifying the origin of key VOCs as either belonging to BC or PRG and identifying which fungal metabolites are being produced exclusively in B + W or B + G SSF. Changes on PC2 were found to be related to the fungal species and explored further with additional PCAs in either B + W or B + G substrates. Furthermore, no significant differences were found between the different strains of each fungus (e.g., AOR or AOB), so they were treated as replicates of the same microorganism (e.g., AO).

Key VOCs of wheat bread are well documented^5,33^, and were identified in our samples. Characteristic compounds identified include products of Maillard reactions, which originate from the non-enzymatic reactions of free amino acids and reducing sugars, mainly glucose^34^, induced by the heat stress in the baking process of bread^35^, such as 2-methylfuran, methylpyrazine and 2,5-dimethylpyrazine, 3-methylbutanal (from leucine) and 2-methylbutanal (from isoleucine) and methylpropanal (from valine). As SSF was performed over surplus bread crusts, which, compared to the bread as a whole, are subject to more heat stress during baking^36^, several of these compounds were identified in unfermented samples.

**Fig. 4:**
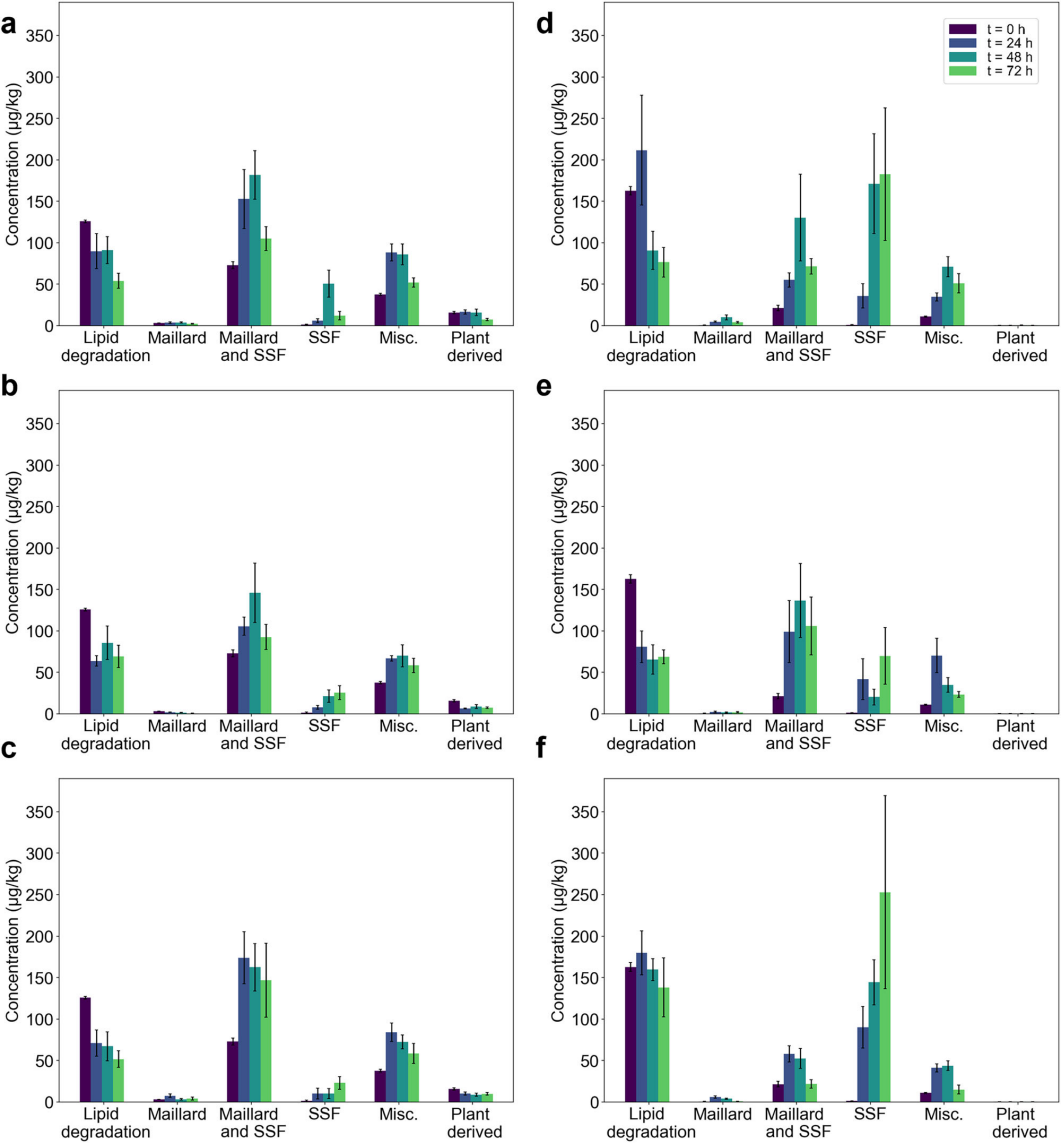
Representative volatile organic compounds (VOC). Approximate mass concentration (μg/kg) of representative VOCs for each origin (lipid degradation: hexanal, Maillard reaction: 2,5-dimethylpyrazine, Maillard reaction and solid-state fermentation (SSF): phenylacetaldehyde, SSF: 2,3-butanediol (sum of both isomers), miscellaneous: benzaldehyde, plant derived: $\beta$-cyclocytral and) at 0, 24, 48, and 72 h of SSF for *Aspergillus oryzae* (AO), *Neurospora intermedia* (NI) and *Rhizopus oligosporus* (RO) in bread crusts + water (B + W) and bread crusts + perennial ryegrass (B + G) substrates. **a** AO in B + G. **b** NI in B + G. **c** RO in B + G. **d** AO in B + W. **e** NI in B + W. **f** RO in B + W. Bars represent the mean value (*n* = 6), and vertical lines the standard error.

Although we have classified the volatiles by their typical formation pathways, for some, there are clearly more than one possible route to formation, as evidenced by the approximate concentration of some compounds related to Maillard reactions, such as 3-methylbutanal, 2-methylbutanal, phenylacetaldehyde and 2-methylpropanal, which also increased in concentration over SSF time, as seen in Fig. 4 with phenylacetaldehyde as an example. This metabolic activity has been reported in SSF of wheat and soybean for koji production with AO^8^ and in SSF of rice bran with RO^37^.

Of all Maillard reaction-related VOCs, Strecker aldehydes were abundant in all samples regardless of the substrate. Across this category, the odor profile of the compounds varied considerably, although common descriptors are related to baked, dairy, sweet, and cooked notes (Table 2). Those with a PC1 value close to 0 (Data S2) were related to both BC and PRG, such as 2-methylbutanal and methylpropanal, and those with a high PC1 value were associated with PRG, such as phenylacetaldehyde, which has been reported in substantial amounts in grasses such as *Festuca sp., Phleum sp*., and *Poa sp*^38^. from the enzymatic decarboxylation of phenylalanine^39^.

Dimethyl sulfide and dimethyl disulfide were abundant sulfurous compounds related to PRG. These compounds, amongst others with green, vegetable, seaweed, and sulfurous undesirable odor notes, were targets of the SSF to reduce the negative aspects of the odor profile of grass. They possess relatively low odor thresholds in water (0.3 and 6 µg/L)^40^, which makes their impact on the overall odor profile significant.

Another common group of compounds was those originating from lipid oxidation. This process can occur at every stage of BC manufacture, in grasses^38^ and during SSF due to the exposure of lipids to oxygen and enzymes (lipases and lipoxygenases) via a free radical mechanism. The resulting hydroperoxides, the main non-volatile intermediates of the reactions, are metabolized into an array of volatile alkanes, aldehydes, ketones, and alcohols^41^. Aside from the enzymes naturally present in wheat flour and in PRG, NI^42^, RO^43^, and AO^44^ have all been reported to produce lipases and lipoxygenases during fermentation, which influences the abundance of these VOCs over time, in some cases reducing their concentration, as can be seen in Fig. 4 with hexanal as an example.

Compounds related to this origin did not possess highly negative PC1 values, likely as the BC were frozen immediately after manufacture to avoid oxidation and only defrosted prior to the start of SSF. Those with a PC1 value close to 0 (Data S2) included hexanal, 2-butanone and 2-heptanone, of which hexanal, the main product of linoleic acid oxidation, has been reported to be one of the most common lipid oxidation indicators in cereal products, meats, nuts, and edible oils^41^. Hexanal made up a substantial proportion of lipid oxidation volatiles in unfermented B + G (140 µg/kg) and B + W (180 µg/kg), which differentially decreased over SSF time based on the conditions, down to 59 µg/kg in RO B + G at 72 h and 73 µg/kg in NI B + W at 48 h. Similarly, the approximate concentration of other lipid oxidation-related VOCs decreased over SSF time, which may positively affect the odor profile of the substrates, as most of these compounds have negative green, fatty, and waxy notes.

Unfermented B + G had a higher diversity of lipid oxidation-related VOCs compared to B + W, with volatiles such as 2-pentenal (19 µg/kg), 1-pentene-3-one (18 µg/kg), 2,6-nonadienal (4 µg/kg), and 2-ethylfuran (43 µg/kg) being present only in B + G. Consequently, most of the compounds in this origin had positive PC1 values (Data S2). No unique VOCs were identified in substantial concentrations in unfermented B + W.

The PRG-related VOCs (Table 2) had the highest positive PC1 scores (Fig. 3a). This group comprises terpenes and sesquiterpenes such as α-phellandrene, linalool, β-bisabolene, and p-cymene. Most of these compounds have pleasant floral, earthy, and herbal notes, are known plant secondary metabolites^45^, and are mostly derived from β-carotene. As β-carotene is not a volatile compound, it was not directly quantified in our tests, but its function in plant photosynthesis and availability in PRG is well known^46^. Amongst the most abundant terpenes identified in unfermented B + G were β-cyclocitral (17 µg/kg) and safranal (10 µg/kg), both closely related and derived from the oxidation of β-carotene, which function as stress signals and play a role in plant communication^47^.

Most of these terpenes and sesquiterpenes did not change notably over SSF time in B + G samples, suggesting that fungal metabolism does not directly interact with them, as shown in Fig. 4 with ${\rm{\beta }}$-cyclocitral as an example. However, other terpenes and sesquiterpenes that were not present in unfermented substrates were uniquely synthesized by RO and NI during SSF in B + W samples, reinforcing the capacity of these fungi to produce VOCs with pleasant aromatic properties. In RO B + W SSF, compounds with distinctive woody and floral notes, α-copaene, α-humelene, α-santalene, caryophyllene, and santalol were synthesized. In NI B + W SSF, compounds with distinctive spice, floral, and fruity notes, β-farnesene, nerolidol, α-bisabolene, β-bisabolene, α-cuparene, β-curcumene, and cubebol were synthesized.

Further analysis of the data required separating the SSF over B + W (Fig. 3b) or B + G (Fig. 3c) substrates, as the diverse, unique compounds in PRG dominated the PCA. Overall, identifying distinct fungi groups, which were also separate from the time 0 substrates, was possible. Additionally, SSF by the same fungal species over different substrates altered the concentration of volatiles differently, suggesting changes in the metabolic pathways used during their growth. In B + W (Fig. 3b) substrates, the approximate total concentration of VOCs increased over time, with the most pronounced rise observed with AO, followed by RO and then NI (Data S3). This difference between fungal species was not related to the diversity of unique compounds but rather the increased metabolism of specific ones. It additionally showed that there are SSF times in which the abundance of particular volatiles was maximized and that this time varied by fungal species.

Overall, the compounds that increased the most over time in B + W substrates were 2-methylbutanal, 3-methylbutanal, and furfural. 2-Methylbutanal and 3-methylbutanal have been reported to be produced by numerous fungal strains^48^, and similarly made available by the increase in free amino acids during SSF by their proteolytic activity over gluten^20^. The highest increase in these compounds was found in AO, while the lowest in NI.

The increase in SSF-related compounds, which were considered outputs of their metabolic activity, differed in each fungus. In AO SSF, 2-methylbutanoic acid and 2-methylpropanoic acid, organic acids with a cheesy and rancid odor, were the most abundant unique volatiles, likely originating from the oxidation of the corresponding aldehydes. Although these volatiles have been reported in the literature alongside their ethyl esters in AO fermentations to produce koji^49^, the esters were not identified in our samples. Aside from acetic acid, which was present in all unfermented substrates and decreased over SSF time, these were the only organic acids identified in our samples.

In NI SSF, multiple unique ethyl esters were identified, such as those originating from decanoic acid, 3-octenoic acid, 4-octenoic acid, octanoic acid, heptanoic acid, and hexanoic acid. Also, esters ethyl *(E)-*2-octenoate and ethyl*-(E)-*2-hexenoate, and alcohols 2-octanol and 3-nonen-1-ol. These compounds have mostly pleasant fruity and ethereal notes. While not all of these volatiles have been previously reported in *Neurospora sp*., there are studies on an acyl coenzyme which can be metabolized by this fungus that allows the synthesis of ethyl hexanoate, ethyl esters, and fruity notes^7,50^.

In RO SSF, unique esters such as ethyl 2-butenoate, citronellyl butanoate, neryl acetate, and α-terpinyl acetate were identified. These compounds have pleasant fruity, floral, and herbal odors and have been identified in multiple strains of *Rhizopus sp*., to the extent that this species has been used in the production of these high-value esters from different agro-industrial wastes^51^.

Additionally, all SSF in B + W had known indicators of fungal growth, such as 2,3-butanediol, 2-methylpropanol, 2-methylbutanol, 3-methylbutanol, and 2-butenal^48^, which increased over time, as can be seen in Fig. 4 with 2,3-butanediol as an example. Other non-reported compounds in SSF were also found, like benzyl alcohol, 2-acetylpyrrole, and 1,3-dioxolanes, with AO synthesizing them in the highest relative concentrations. This last group of compounds is cyclic acetals formed from the condensation of carbonyl groups, widely available in the form of aldehydes and ketones, and a diol, of which only 2,3-butanediol was identified in our samples. They have been identified in some foods and plant material and characterized as having pleasant fruity notes^52^ but are mostly studied for their properties as solvents. Even though it is not common to find them reported in the literature, they comprised a critical proportion of the total abundance of VOCs in fermented samples.

A notable compound found missing from all samples commonly reported as a volatile in SSF was ethanol, which was likely lost to evaporation in the drying process, but must have been present, as it is necessary for most of the esterification reactions which yielded a variety of ethyl esters. A broader spectrum of volatile fatty acids, including linear saturated carboxylic acids from butanoic to tetradecanoic acid, was also expected to be present in all SSF samples^8,13^, but likely went undetected due to their higher polarity and lower volatility. Lastly, informal olfactory assessments of the samples revealed a strong aroma reminiscent of molasses and sugarcane, potentially attributable to the presence of furanones or pyranones such as sotolone, which is a highly odor-active sugar degradation compound known to occur in molasses but also formed during fermentation. It was not detected in this analysis, and alternative analytical methods are required to detect this compound at low concentrations.

In B + G (Fig. 3c), the approximate total concentration of VOCs did not change meaningfully over SSF time, but the relative concentration of specific compounds did. Furfural, 2-methylbutanal, and 3-methylbutanal, Maillard reaction compounds which have also been reported as VOCs of PRG, were the most abundant VOCs in unfermented B + G. Contrary to B + W SSF, these compounds decreased over time, and direct derivatives such as 3-methylbutanol increased.

The unique SSF volatiles synthesized in B + G also changed considerably, due to both the change in metabolic pathways needed to grow in this substrate and the fact that NI and RO were not able to grow as fast (Fig. 1). Some of the unique volatiles synthesized in B + W SSF were not found in these samples, such as the terpenes and sesquiterpenes in RO and NI, most of the ethyl esters in NI and the organic acids in AO. Instead, different unique volatiles were formed in AO and RO. In AO SSF, 5-methyl-2(5*H*)-furanone, with sweet and maple notes, and 2-methylbutyl butanoate, with fruity notes, were identified. In RO SSF, 2-phenylethyl propanoate and 2-phenylethyl acetate, with fruity and sweet notes, and 2-methylpropanol, with ethereal and fermented notes, were identified. There was also a substantial increase in the abundance of SSF-related VOCs common across all fungi, in addition to those already mentioned for B + W, such as phenylethyl alcohol, 4-ethenyl-2-methoxyphenol, and 2-pyrrolecarboxaldehyde, with diverse odor notes.

SSF over B + G decreased the concentration of volatiles with unpleasant grassy, vegetable, sulfurous, seaweed, and rancid odors, with AO reducing their relative abundance the most, followed by NI and lastly RO. Amongst the most decreased volatiles were hexanal, dimethyl sulfide, 2-pentylfuran, 2-hexenal, 1-penten-3-one, and dimethyl disulfide. Overall, the decrease was significant when comparing the SSF to the unfermented substrates, but not when comparing the different fungi between each other or over SSF time, which indicates that after 24 h, all the fungi had metabolized most of these compounds similarly. In the case of dimethyl disulfide, SSF with RO and NI resulted in the greatest reduction, with a clear time-dependent effect whereby fermentation for 48 and 72 h yielded the lowest abundance.

Overall, this study shows the different chemical pathways that occur under different fermentation conditions and the capacity of different fungi to decrease the concentration of volatile compounds with unfavorable odor notes, which are characteristic of forage crops, as well as the synthesis of others with diverse pleasant notes. Interestingly, we identified a diverse set of volatiles not previously reported in SSF with these fungi, some of which were common across all species. While different strains of the same fungal species did not yield statistically significant differences, each species synthesized unique compounds, suggesting that there is no single ‘optimal’ strain for this combination of substrates, but rather diverse odor profiles that could be tailored to different novel food applications. These fermented products have practical potential as alternative protein sources produced from locally sourced materials and food waste, contributing to increased circularity in food systems while providing new protein-rich food ingredients. Future research should aim to optimize the inclusion levels of forage crop protein in SSF substrates to maximize nutritional value while mitigating undesirable odor notes.

## Methods

### Raw materials

For SSF, phosphoric acid, potato dextrose agar (PDA), and Tween 80 were purchased from Merck Life Science UK Ltd (Dorset, UK). Bread crusts (BC) from surplus wheat flour loaf bread were provided by Bradgate Bakery (a division of Samworth Brothers Ltd, Leicestershire, UK) and manually cut to approximately 1 × 1 × 1 cm cubes.

For solid-phase microextraction (SPME) gas chromatography-mass spectrometry (GC-MS), a divinylbenzene/carboxen/polydimethylsiloxane (DVB/CAR/PDMS) fiber was purchased from Supelco (Nottingham, UK), and a J&W DB-5MS column was purchased from Agilent Technologies LDA (Stockport, UK). To calculate authentic linear retention indices (LRI) in the DB-5MS column, 2,3-dimethylpyrazine, 2,5-dimethylpyrazine, 3-methylfuran, ethylpyrazine, 2-furfural, and methylpyrazine were purchased from Acros Organics (Thermo Fisher Scientific) (Geel, Belgium). 3-Methylbutanal was purchased from Alfa Aesar (Thermo Fisher Scientific) (Ward Hill, Massachusetts, USA). 3,6-Dimethyl-2-ethylpyrazine was purchased from AromaLAB GmbH (Planegg, Germany). 2-Pentylfuran was purchased from Avocado Research Chemicals Ltd (Heysham, UK). 2,6-Diethylpyrazine was purchased from Dalgety plc (Genus plc) (Hampshire, UK). *(E)*-2-Decenal, *(E)-*2-heptenal, α-copaene, and p-xylene were purchased from Fluka (Honeywell) (New Jersey, USA). 2-Phenethyl acetate, eugenol, and ethyl hexanoate were purchased from Givaudan SA (Vernier, Switzerland). Hexanol, 2-heptanone, ethyl decanoate, ethyl octanoate, 2-methylbutyl butanoate, and 2-hydroxy-3-methyl-2-cyclopenten-1-one were purchased from International Flavors & Fragrances Inc. (New York, USA). Cubebol, α-santalene, *(Z)-*α-bisabolene, and β-bisabolene were supplied by Isobionics B.V. (BASF) (Geleen, Netherlands). 1-Penten-3-ol, 3-methyl-2-thiophenecarboxaldehyde, and 5-methyl-2-thiophenecarboxaldehyde were purchased from Lancaster Synthesis Ltd (Thermo Fisher Scientific) (Ward Hill, Massachusetts, USA). α-Terpinyl acetate, citronellyl butanoate, ethyl lactate, geranyl butanoate, 1,2-dimethoxy-4-prop-1-enylbenzene, neryl acetate, and santalol were purchased from Mane Ltd UK (Derby, UK). 2-Isopropylpyrazine and 2,3-octanedione were purchased from Merck Life Science UK Ltd. 2-Ethylfuran, 2-ethenylfuran, *(E)-*2-pentenal, 2-furanmethanol, 2-methylfuran, and 1-pentene-3-one were purchased from Oxford Chemicals Ltd (Hartlepool, UK). *(Z)-*Nerolidol was purchased from PFW Aroma Chemicals B.V. (Barneveld, Netherlands). 5-Methyl-2-phenyl-2-hexenal was purchased from Riverside Organics Ltd (Northwich, UK). 3-Methyl-2-butenal, 3-methylbutanol, 2,3-butanedione, 2-methylbutanoic acid, 3-methylbutanoic acid, phenylacetaldehyde, 2-methylbutanal, dimethyl sulfide, methional, phenylethyl alcohol, 2-hexenyl acetate, *(E)-*2-nonenal, 2-acetylfuran, 1-(2-furanylmethyl)-1*H*-pyrrole, 1-ethylpyrrole, butanol, 2-methylbutanol, 2-pyrrolecarboxaldehyde, octanol, 2-methylpropanol, β-cyclohomocitral, *(E,E)-*2,6-nonadienal, *(E,Z)-*2,6-nonadienal, 2-acetylpyridine, 2-acetylpyrrole, 2-butenal, 2-ethyl-5-methylpyrazine, 2-hexen-1-ol, 4-ethenyl-2-methoxyphenol, 2-methyl-2-cyclopentenone, *(E)-*2-octenal, *(E)-*2-undecenal, *(Z)-*3-hexen-1-ol, *(Z)-*3-hexenal, 3-hydroxybutanone, 3-methyl-2-butanone, 2-butanone, *(Z)-*4-heptenal, 4-methyl-2-(2-methylpropyl)-1,3-dioxolane, 4-ethenylphenol, 6-methyl-5-heptene-2-one, 5-methyl-2(3*H*)-furanone, 5-methyl-2(5*H*)-furanone, 6,10-dimethyl-5,9-undecadien-2-one, 6-methyl-2-heptanone, α-humulene, α-phellandrene, α-terpineol, *(E)-*β-farnesene, benzyl alcohol, β-cyclocitral, caryophyllene, dimethyl disulfide, ethyl acetate, hexanal, linalool, nonane, p-cymene, pentanal, methylpropanal, 2-methylpropanoic acid, propyl propanoate, safranal, terpinolene, heptanol, 2-methyl-2-butenal, 2-octanol, α-cyclocitral, d-limonene, ethyl 2-butenoate, 1,1,6-trimethyl-1,2-dihydronaphthalene, and *(E)-*5-methyl-2-isopropyl-2-hexenal were purchased from Sigma-Aldrich (Merck Ltd) (Missouri, USA). Nonanal was purchased from Synergy Flavors Inc. (Illinois, USA). Pentanol, 2,3-butanediol, *(E)-*2-hexenal, acetic acid, benzaldehyde, and ethyl *(E)-*2-hexenoate were purchased from Tokyo Chemical Industry Co., Ltd. (Tokyo, Japan). 2,3-Pentanedione was purchased from Thermo Fisher Scientific Ltd (Paisley, UK). 1-(3-Methylbutyl)pyrrole, 4,5-dimethyl-2-isopropyl-1,3-dioxolane, and 4,5-dimethyl-2-isobutyl-1,3-dioxolane were synthesized at Reading University, Reading, UK^53,54^.

### Perennial ryegrass harvesting and pilot-scale processing

PRG was seeded in 2022 in a farmed experimental plot at Aberystwyth University in Aberystwyth, UK, and harvested in June 2023. Freshly harvested PRG (1.5 metric ton) was screw pressed at pilot scale in a CP-10 screw press (Vincent corporation, FL, US) to obtain 610 L of grass juice (GJ) with a pH of 6.00, and frozen at −20 °C in 3 L aliquots to avoid the growth of undesired microorganisms. The resulting GJ had a DM content of 5.13% and a crude protein (CP) content of 16.0% DM. GJ (555 L) was clarified using a clarifying decanter SCE 205 (GEA Mechanical Equipment UK Ltd, UK) at 5590 rpm, with an internal speed differential of 12 rpm, to obtain 510 L of clarified GJ with a DM content of 0.99%, subsequently adjusted to pH 3.5 with H_3_PO_4_. The clarified GJ was centrifuged in a Scout separator SSE 10 (GEA Mechanical Equipment UK Ltd, UK) at 21,000 g to obtain 25 kg of wet protein-rich precipitate with a DM content of 21.9%, which was freeze-dried, achieving a grass solid (GS) with a final DM content of 85.0% and CP content of 40.1% DM.

### Microorganism and inoculum preparation

The fungal strains *Rhizopus oligosporus* (Tempeh) (ROT), *Aspergillus oryzae* (Red Rice Koji) (AOR), and *Aspergillus oryzae* (Barley Koji) (AOB) were purchased from fermentationculture.eu. *Rhizopus oligosporus* (DSMZ 1964) (ROD) and *Neurospora intermedia* (DSMZ 1265) (NID) were purchased from the German Collection of Microorganisms and Cell Culture, Leibniz Institute, Germany. *Neurospora intermedia* (CBS 131.92) (NIC) was purchased from the Westerdijk Fungal Biodiversity Institute, the Netherlands.

Fungal spores were preserved in 10% glycerol solution at −80 °C and in PDA plates at 32 °C. After 5 days of growth at 32 °C, spores were harvested from PDA plates in 50 mL sterile distilled water (0.03% Tween 80) and counted in a Neubauer chamber to approximately 1 × 10^7^ spores/mL, and SSFs were inoculated at 2 × 10^5^ spores/g substrate.

### Solid-state fermentation

SSF experiments were conducted in sterile 20 × 8 × 5 cm aluminum trays according to Sandoval et al.^20^. Briefly, 100 g of BC, with an initial CP content of 15.9 ± 0.2% DM, were either adjusted to a moisture content of 56% (v/w DM) with sterile distilled water (W) to make up the BC + water (B + W) substrate or supplemented with GS to a CP content of 24.5 ± 0.1% and adjusted to a moisture content of 56% with GJ to make up the BC + PRG (B + G) substrate. Each fungal strain was inoculated into the B + W and B + G substrates and incubated for 24, 48, or 72 h at pH 3.5 (adjusted with H_3_PO_4_) and 32 °C. After the fermentation, the fermented and non-fermented samples of the B + W and B + G substrates were dried in a vacuum oven at 45 °C, milled to a fine powder, and then stored at 4 °C for further analysis. Each experimental condition was done in biological triplicate, giving a total of 114 experiments.

### Chemical composition analysis

The moisture content of the samples was determined according to the AOAC method 930.15^55^. Crude protein content was determined using the Dumas combustion method in a Vario MAX Cube CN analyzer (Elementar, Stockport, UK). A conversion factor of 6.25 was used to convert total nitrogen to crude protein content.

### Solid phase microextraction

The VOCs were extracted by SPME using a CTC-CombiPal auto-sampler MS (Agilent Technologies, Stockport, UK) equipped with a DVB/CAR/PDMS fiber. For each experiment, 1 g of dry sample was mixed in an 18 mL glass vial with 3 mL of deionized water (to allow use of an internal standard). The internal standard (8 µL of a 100-µg/L aqueous solution of 2-isopropylpyrazine) was added, and the vial fitted with a screw cap and a PTFE-lined septum. After equilibration at 50 °C for 20 min, the SPME fiber was exposed to the headspace above the sample for 20 min at 50 °C. Each biological replicate was measured once.

### GC-MS analysis of SPME extracts

After extraction, the SPME device was inserted into the injection port of a 7890 A GC equipped with a J&W DB-5MS column coupled to a 5975 C inert XL EI/CI MSD triple axis MS (Agilent Technologies, Stockport, UK). The contents of the SPME fiber were desorbed for 20 min at 40 °C in a split/splitless injection port in splitless mode. The injector and detector temperatures were maintained at 280 and 250 °C, respectively. After desorption, the oven was maintained at 40 °C for 2 min and then the temperature was raised at 4 °C/min to 250 °C, and held for 1 min. Helium was the carrier gas, and the flow rate was 2.0 mL/min. Mass spectra were recorded in the electron-impact mode at an ionization voltage of 70 eV and source temperature of 200 °C, with a scan range of 29–500 m/z and a scan time of 0.69 s. The data were controlled and stored by the HP G1034C ChemStation data system.

### Volatile compound identification and quantification

VOCs were identified by comparison of each mass spectrum with spectra from authentic compounds analyzed at Reading University, or spectra from the NIST/EPA/NIH Mass Spectral database (Version 2.0 g, 2011). To confirm the identification, the LRI was calculated for each VOC, using the retention times of a homologous series of C6–C20 n-alkanes and by comparing the LRI with those of authentic compounds analyzed under similar conditions. The approximate quantification of VOCs was calculated from GC peak areas by comparing with the peak area of the 2-isopropylpyrazine standard, using a response factor of 1.

The identified VOCs were categorized by functional chemical groups (as either alcohols, aldehydes, esters, furans, ketones, organic acids, phenols, pyrazines, pyridines, pyrroles, sulfur compounds, terpenes, dioxolanes, or others) and by likely source of origin (as either lipid degradation, Maillard reaction, plant derived, SSF, or miscellaneous). Their odor descriptors were identified by doing blind smelling trials of 1% solutions of authentic compounds in ethanol if the compound was available (Table 2). Otherwise, the odor descriptors were taken as reported in The Good Scents Company database^26^ (Data S1).

### Statistical analysis

Multivariate analysis of variance (MANOVA) was used to analyze the approximate concentration of all VOCs in all samples. Two-tailed one-way analysis of variance (ANOVA) followed by Tukey’s HSD post hoc test was used to analyze the crude protein contents and the approximate concentration of key VOCs. The tests were done with a statistical significance level $\alpha$ of 0.05.

To analyze the correlation between the identified volatiles, the approximate mass concentration of the VOCs in each sample was compared via principal component analysis (PCA) using the scikit-learn library in Python v3.11. PCA was performed on the covariance matrix after transforming the data (mean = 0 and variance = 1) to account for the significant variability in the order of magnitude in the concentration of some compounds. A total of *n* = 5 components were analyzed, and results are shown for principal components (PC) PC1, and PC2.

## Supplementary information

Supplementary Information

Data S1

Data S2

Data S3

## Data Availability

The data supporting the findings reported herein are available on request from the corresponding author.

## References

[1] López-Gómez, J. P., Manan, M. A. & Webb, C. in *Food Industry Wastes (Second Edition)* (eds Maria, R. K. & Colin, W.) 135–161 (Academic Press, 2020).

[2] Narisetty, V. et al. Recycling bread waste into chemical building blocks using a circular biorefining approach. *Sustain. Energy Fuels*. **5**, 4842–4849 (2021). 34604539 10.1039/d1se00575h PMC8477656

[3] Hellwig, C. et al. Household fermentation of leftover bread to nutritious food. *Waste Manag.* **150**, 39–47 (2022). 35792440 10.1016/j.wasman.2022.06.038

[4] Gmoser, R. et al. From stale bread and brewers spent grain to a new food source using edible filamentous fungi. *Bioengineered*. **11**, 582–598 (2020). 32449450 10.1080/21655979.2020.1768694 PMC8291841

[5] Pétel, C., Onno, B. & Prost, C. Sourdough volatile compounds and their contribution to bread: A review. *Trends Food Sci. Technol.* **59**, 105–123 (2017).

[6] Frasse, P., Lambert, S., Richard-Molard, D. & Chiron, H. The influence of fermentation on volatile compounds in French bread dough. *LWT Food Sci. Technol.* **26**, 126–132 (1993).

[7] Yamauchi, H. et al. Production and application of a fruity odor in a solid-state culture of *Neurospora sp.* using pregelatinized polished rice. *Agric. Biol. Chem.* **53**, 2881–2886 (1989).

[8] Han Sol, S. et al. Evaluating the headspace volatolome, primary metabolites, and aroma characteristics of koji fermented with Bacillus amyloliquefaciens and *Aspergillus oryzae*. *J. Microbiol. Biotechnol.* **28**, 1260–1269 (2018). 30301311 10.4014/jmb.1804.04017

[9] Chen, X. et al. Quantitative analyses for several nutrients and volatile components during fermentation of soybean by Bacillus subtilis natto. *Food Chem.* **374**, 131725 (2022). 35021579 10.1016/j.foodchem.2021.131725

[10] Zhang, D., Huang, Y., Fan, X. & Zeng, X. Effects of solid-state fermentation with *Aspergillus cristatus* (MK346334) on the dynamics changes in the chemical and flavor profile of dark tea by HS-SPME-GC–MS, HS-GC-IMS and electronic nose. *Food Chem.* **455**, 139864 (2024). 38833862 10.1016/j.foodchem.2024.139864

[11] Mantzouridou, F. T., Paraskevopoulou, A. & Lalou, S. Yeast flavour production by solid state fermentation of orange peel waste. *Biochem. Eng. J.* **101**, 1–8 (2015).

[12] Tu, J. et al. Solid state fermentation by Fomitopsis pinicola improves physicochemical and functional properties of wheat bran and the bran-containing products. *Food Chem.* **328**, 127046 (2020). 32470773 10.1016/j.foodchem.2020.127046

[13] Astuti, R. D. et al. The volatile compounds and aroma description in various *Rhizopus oligosporus* solid-state fermented and nonfermented rice bran. *Fermentation*. **8**, 120 (2022).

[14] Fariaszewska, A. et al. Mild drought stress-induced changes in yield, physiological processes and chemical composition in Festuca, Lolium, and Festulolium. *J. Agron. Crop Sci.* **203**, 103–116 (2017).

[15] Stødkilde, L., Damborg, V. K., Jørgensen, H., Lærke, H. N. & Jensen, S. K. Digestibility of fractionated green biomass as protein source for monogastric animals. *Animal* **13**, 1817–1825 (2019). 30774050 10.1017/S1751731119000156

[16] Penrose, B., Lovatt, J. A., Palmer, S., Thomson, R. & Broadley, M. R. Revisiting variation in leaf magnesium concentrations in forage grasses for improved animal health. *Plant Soil*. **457**, 43–55 (2020).

[17] Hamacher, M., Malisch, C. S., Reinsch, T., Taube, F. & Loges, R. Evaluation of yield formation and nutritive value of forage legumes and herbs with potential for diverse grasslands due to their concentration in plant specialized metabolites. *Eur. J. Agron.* **128**, 126307 (2021).

[18] Zhang, W., Grimi, N., Jaffrin, M., Ding, L.-H. & Tang, B. A. Short review on the research progress in alfalfa leaf protein separation technology. *J. Chem. Technol. Biotechnol* **92**, 2894–2900 (2017).

[19] Mumbi, A. W., Pittson, H., Vriesekoop, F. & Kurhan, S. Consumer acceptance of grass-derived ingredients in the UK: a cross-sectional study. *Sustainability*. **16**, 7161 (2024).

[20] Sandoval, J. F., Gallagher, J., Rodriguez-Garcia, J., Whiteside, K. & Bryant, D. N. Improved nutritional value of surplus bread and perennial ryegrass via solid-state fermentation with *Rhizopus oligosporus*. *npj Sci. Food* **8**, 95 (2024). 39550376 10.1038/s41538-024-00338-y PMC11569167

[21] Worku, A. et al. Aroma profile, microbial and chemical quality of ensiled green forages mixtures of winter cereals and Italian ryegrass. *Agriculture* **11**, 512 (2021).

[22] Morgan, M. E. & Pereira, R. L. Volatile constituents of grass and corn silage. i. steam distillates1. *J. Dairy Sci.* **45**, 457–466 (1962).

[23] Kagan, I. A. Soluble phenolic compounds of perennial ryegrass (Lolium perenne L.): potential effects on animal performance, and challenges in determining profiles and concentrations. *Anim. Feed Sci. Technol.* **277**, 114960 (2021).

[24] Senoussi, A. et al. Botanical composition and aroma compounds of semi-arid pastures in Algeria. *Grass Forage Sci.* **76**, 282–299 (2021).

[25] Hafner, S. D. et al. Emission of volatile organic compounds from silage: compounds, sources, and implications. *Atmos. Environ.* **77**, 827–839 (2013).

[26] TGSC. *The Good Scents Company Information System*, <https://www.thegoodscentscompany.com/> (2025).

[27] Try, S., Voilley, A., Chunhieng, T., De-Coninck, J. & Waché, Y. Aroma compounds production by solid state fermentation, importance of in situ gas-phase recovery systems. *Appl. Microbiol. Biotechnol.* **102**, 7239–7255 (2018). 29938320 10.1007/s00253-018-9157-4

[28] Kaminski, E., Stawicki, S. & Wasowicz, E. Volatile flavor compounds produced by molds of *Aspergillus*, *Penicillium*, and fungi imperfecti. *Appl. Microbiol.* **27**, 1001–1004 (1974). 16349989 10.1128/am.27.6.1001-1004.1974 PMC380197

[29] Gmoser, R., Lennartsson, P. R. & Taherzadeh, M. J. From surplus bread to burger using filamentous fungi at bakeries: techno-economical evaluation. *Clean. Environ. Syst.* **2**, 100020 (2021).

[30] McCance, R. A. & Widdowson, E. M. *McCance and Widdowson’s the Composition of Foods Integrated Dataset 2021*. (Public Health England, 2021).

[31] FAO Dietary protein quality evaluation in human nutrition. *FAO Food Nutr. Pap.* **92**, 1–66 (2011). 26369006

[32] Schnürer, J., Olsson, J. & Börjesson, T. Fungal volatiles as indicators of food and feeds spoilage. *Fungal Genet. Biol.* **27**, 209–217 (1999). 10441446 10.1006/fgbi.1999.1139

[33] Kirchhoff, E. & Schieberle, P. Determination of key aroma compounds in the crumb of a three-stage sourdough rye bread by stable isotope dilution assays and sensory studies. *J. Agric. Food Chem.* **49**, 4304–4311 (2001). 11559129 10.1021/jf010376b

[34] Wieser, H., Koehler, P. & Scherf, K. A. Chemistry of wheat gluten proteins: qualitative composition. *Cereal Chem.* **100**, 23–35 (2023).

[35] Martins, S. I. F. S., Jongen, W. M. F. & van Boekel, M. A. J. S. A review of Maillard reaction in food and implications to kinetic modelling. *Trends Food Sci. Technol.* **11**, 364–373 (2000).

[36] Çelik, E. E. & Gökmen, V. Formation of Maillard reaction products in bread crust-like model system made of different whole cereal flours. *Eur. Food Res. Technol.* **246**, 1207–1218 (2020).

[37] Mar’Atun Nadhifah, A. et al. The volatile compounds and aroma profile of some pigmented rice brans after fermentation. *Curr. Res. Nutr. Food Sci.* **10**, 145–170 (2022).

[38] Tava, A. et al. Volatile constituents of Festuca nigrescens, Phleum alpinum and Poa alpina from N.W. Italian alpine pastures. *Nat. Prod. Commun.* **6**, 1934578X1100600124 (2011). 21366056

[39] Kaminaga, Y. et al. Plant phenylacetaldehyde synthase is a bifunctional homotetrameric enzyme that catalyzes phenylalanine decarboxylation and oxidation. *J. Biol. Chem.* **281**, 23357–23366 (2006). 16766535 10.1074/jbc.M602708200

[40] Fors, S. Sensory properties of volatile Maillard reaction products and related compounds, a literature review. The Maillard reaction in foods and nutrition. *ACS Symp. Ser.* **215**, 185–286 (1983).

[41] Azarbad, M. H. & Jeleń, H. Determination of hexanal—an indicator of lipid oxidation by static headspace gas chromatography (SHS-GC) in fat-rich food matrices. *Food Anal. Methods* **8**, 1727–1733 (2015).

[42] Kumbhare, P. Activity assay of the enzymes amylase, protease and lipase produced by N. Intermedia MTCC 1230 and R. Oligosporus MTCC 556 during peanut press cake fermentation. *Int. J. Res. Biosci. Agric. Technol.* **2**, 183–187 (2015).

[43] Nahas, E. Control of lipase production by *Rhizopus oligosporus* under various growth conditions. *Microbiology* **134**, 227–233 (1988).

[44] Sugio, A., Østergaard, L. H., Matsui, K. & Takagi, S. Characterization of two fungal lipoxygenases expressed in *Aspergillus oryzae*. *J. Biosci. Bioeng.* **126**, 436–444 (2018). 29805113 10.1016/j.jbiosc.2018.04.005

[45] Araniti, F., Sánchez-Moreiras, A. M., Graña, E., Reigosa, M. J. & Abenavoli, M. R. Terpenoid trans-caryophyllene inhibits weed germination and induces plant water status alteration and oxidative damage in adult Arabidopsis. *Plant Biol.* **19**, 79–89 (2017). 27173056 10.1111/plb.12471

[46] McCurdy, J. D., McElroy, J. S., Kopsell, D. A., Sams, C. E. & Sorochan, J. C. Effects of mesotrione on perennial ryegrass (Lolium perenne L.) carotenoid concentrations under varying environmental conditions. *J. Agric. Food Chem.* **56**, 9133–9139 (2008). 18788815 10.1021/jf801574u

[47] Havaux, M. β-Cyclocitral and derivatives: emerging molecular signals serving multiple biological functions. *Plant Physiol. Biochem.* **155**, 35–41 (2020). 32738580 10.1016/j.plaphy.2020.07.032

[48] Jampílek, J. & Kráľová, K. in *Fungal Secondary Metabolites* (eds Kamel, A. A. & Heba, I. M.) 399-426 (Elsevier, 2024).

[49] Li, J. et al. Untargeted metabolomic profiling of *Aspergillus sojae* 3.495 and *Aspergillus oryzae* 3.042 fermented soy sauce koji and effect on moromi fermentation flavor. *LWT* **184**, 115027 (2023).

[50] Yamauchi, H. et al. Cell-free synthesis of ethyl hexanoate by extract from *Neurospora* sp., containing a novel acyl coenzyme A: alcohol acyltransferase. *Agric. Biol. Chem.* **53**, 821–825 (1989).

[51] Ghosh, B. & Ray, R. R. Current commercial perspective of *Rhizopus oryzae*: a review. *J. Appl. Sci.* **11**, 2470–2486 (2011).

[52] Samsudin, M. W., Rongtao, S. & Said, I. M. Volatile compounds produced by the reaction of leucine and valine with glucose in propylene glycol. *J. Agric. Food Chem.* **44**, 247–250 (1996).

[53] Sullivan, R. C. *The Flavour of Cooked Cheese.* Doctor of Philosophy thesis, (University of Reading, 2023).

[54] Elmore, J. S., Parker, J. K., Halford, N. G., Muttucumaru, N. & Mottram, D. S. Effects of plant sulfur nutrition on acrylamide and aroma compounds in cooked wheat. *J. Agric. Food Chem.* **56**, 6173–6179 (2008). 18624444 10.1021/jf0730441

[55] AOAC. *Official Methods of Analysis*. 18 edn, (Association of Official Analytical Chemists, 2010).

