# Supplementary material for: Transforming the odor profile of perennial ryegrass protein and surplus bread crusts through solid-state fermentation
Source: NPJ Sci Food. 2026 Apr 24;10:197. doi: 10.1038/s41538-026-00831-6 (PMC13282373; doi:10.1038/s41538-026-00831-6)
Supplement: Supplementary file 1 — Supplementary Information [file 41538_2026_831_MOESM1_ESM.pdf]

Supplementary data for **Transforming the Odor Profile of Perennial Ryegrass Protein and Surplus Bread Crusts Through Solid-State Fermentation**

Data S1. Identification ions, linear retention index (LRI), functional group category, source of origin category and odor descriptors of volatile compounds detected by SPME GC-MS in solid-state fermented (SSF) surplus bread crusts and perennial ryegrass with Aspergillus oryzae, Neurospora intermedia and Rhizopus oligosporus.

Data S2: Principal components from principal component analysis (PCA) on the approximate mass concentration of volatile compounds detected by SPME GC-MS in solid-state fermented (SSF) surplus bread crusts and perennial ryegrass with Aspergillus oryzae, Neurospora intermedia and Rhizopus oligosporus over SSF time.

Data S3: Approximate mass concentration of volatile compounds detected by SPME GC-MS in solid-state fermented (SSF) surplus bread crusts and perennial ryegrass with Aspergillus oryzae, Neurospora intermedia and Rhizopus oligosporus.
